# Prioritization of copper for the use in photosynthetic electron transport in developing leaves of hybrid poplar

**DOI:** 10.3389/fpls.2015.00407

**Published:** 2015-06-03

**Authors:** Muhammad Shahbaz, Karl Ravet, Graham Peers, Marinus Pilon

**Affiliations:** Department of Biology, Colorado State University, Fort CollinsCO, USA

**Keywords:** plastocyanin, photosynthesis, copper deficiency, prioritization, polyphenol oxidase, superoxide dismutase, Cu-miRNA, poplar

## Abstract

Plastocyanin (PC) is an essential and abundant copper (Cu) protein required for photosynthesis in higher plants. Severe copper deprivation has the potential to cause a defect in photosynthetic electron transport due to a lack in PC. The Cu-microRNAs, which are up-regulated under Cu deficiency, down-regulate the expression of target Cu proteins other than PC, cytochrome*-c* oxidase and the ethylene receptors. It has been proposed that this mechanism saves Cu for PC maturation. We aimed to test how hybrid poplar, a species that has capacity to rapidly expand its photosynthetically active tissue, responds to variations in Cu availability over time. Measurement of chlorophyll fluorescence after Cu depletion revealed a drastic effect on photosynthesis in hybrid poplar. The decrease in photosynthetic capacity was correlated with a reduction in PC protein levels. Compared to older leaves, PC decreased more strongly in developing leaves, which also lost more photosynthetic electron transport capacity. The effect of Cu depletion on older and more developed leaves was minor and these leaves maintained much of their photosynthetic capacity. Interestingly, upon resupply of Cu to the medium a very rapid recovery of Cu levels was seen in the younger leaves with a concomitant rise in the expression and activity of PC. In contrast, the expression of those Cu proteins, which are targets of microRNAs was under the same circumstances delayed. At the same time, Cu resupply had only minor effects on the older leaves. The data suggest a model where rapid recovery of photosynthetic capacity in younger leaves is made possible by a preferred allocation of Cu to PC in younger leaves, which is supported by Cu-microRNA expression.

## Introduction

Copper (Cu) is a redox-active transition metal essential for plant growth and development. The reversible oxidation-reduction makes Cu very valuable as a cofactor in several metalloproteins. Cu-proteins in plants include PC, cytochrome*-c* oxidase, the ethylene receptors, copper/zinc superoxide dismutase (Cu/ZnSOD), ascorbate oxidase, amine-oxidase, laccase (LAC), plantacyanin and polyphenol oxidase (PPO; for a review see: Cohu and Pilon, 2010). Three of the most abundant plant Cu proteins are found in the chloroplasts: PC, Cu/ZnSOD, and PPO. PC is a single Cu atom containing protein located in the thylakoid lumen that is essential for photosynthetic electron transport in plants (Weigel et al., 2003). Cu/ZnSOD, which contains one Cu and one Zn atom per monomer, is the main Cu protein in the stroma with another major Cu/ZnSOD isoform found in the cytosol. Cu/ZnSODs are thought to play a role in reactive oxygen species metabolism. However, an *Arabidopsis* mutant, which has undetectable Cu/ZnSOD activity in the chloroplasts and cytosol was phenotypically the same as a wild-type, even under stress (Cohu et al., 2009). Therefore, the exact biological role of these very conserved Cu proteins is still unclear. Since plants also have FeSOD in the plastids it is possible that Cu/ZnSOD and FeSOD functions are redundant. The laccases form a diverse group of secreted enzymes that belong to the multicopper oxidase family. Recent evidence links specific laccases to lignin formation both in *Arabidopsis* (Berthet et al., 2011; Zhao et al., 2013) and in poplar (Lu et al., 2013). Plantacyanin is a small, secreted, Cu-protein that is structurally similar to PC. In *Arabidopsis* plantacyanin is thought to have a role in reproduction perhaps by mediating pollen tube growth (Dong et al., 2005). PPO is a binuclear Cu protein found in the thylakoid lumen. PPO catalyzes the conversion of monophenols to ortho-diphenol and ortho-dihydroxyphenols to ortho-quinones that appears as black or brown pigment deposits in fruits and vegetative plant issues (Mayer, 2006). PPO is found in several plants including poplar (Constabel et al., 2000; Ravet et al., 2011), but missing in *Arabidopsis thaliana* (Schubert et al., 2002). PPO was proposed to function in defense against herbivory (Wang and Constabel, 2004).

Cu homeostasis is regulated in *Arabidopsis* via the conserved transcription factor SPL7 (squamosa promoter binding protein-like 7; Yamasaki et al., 2009; Bernal et al., 2012). Under Cu deficiency, SPL7 up regulates the expression of cell surface COPT proteins and reductases which function in Cu uptake (Bernal et al., 2012). In addition, under deficiency, SPL7 mediates the down-regulation of several Cu proteins including Cu/ZnSODs, laccases, and plantacyanin by affecting turnover of transcripts, which are targets of the Cu-microRNAs (Abdel-Ghany and Pilon, 2008; Burkhead et al., 2009). These Cu microRNAs are themselves up-regulated by SPL7 (Yamasaki et al., 2007, 2009; Abdel-Ghany and Pilon, 2008; Bernal et al., 2012; Zhang et al., 2014). The Cu-microRNAs are called miR397 (targets LACs), miR398 (targets Cu/ZnSOD and the Cu chaperone for SOD), miR408 [targets LACs and plantacyanin a secreted protein; and miR1444 (targets PPO, absent in *Arabidopsis*; Abdel-Ghany and Pilon, 2008; Ravet et al., 2011)]. There is one locus for miR408 but there are several loci for each of miR397, miR398, and miR1444 (Ravet et al., 2011). MiR397, miR398, and miR408 are among the most conserved microRNAs in plants (Axtell and Bowman, 2008). As a Cu-economy hypothesis it was proposed that in Cu deprived conditions plants adjust the allocation of Cu for the efficient utilization of the available Cu by down-regulation of potentially redundant Cu containing proteins via Cu-miRNAs (Yamasaki et al., 2007; Burkhead et al., 2009; Ravet et al., 2011). However, it has become clear that SPL7 mediates the expression of a larger set of genes and that SPL7 affects many of its targets in combined action with other transcription factors such as HY5 (Zhang et al., 2014), which mediates responses to light. The observation of combinatorial control suggests that Cu homeostasis is integrated with environmental and developmental cues.

In natural soils and in agriculture deficiency due to low Cu bioavailability can be a problem (Burkhead et al., 2009). Typical symptoms of Cu deficiency include stunted growth due to defects in apical meristems, chlorosis, curling of leaf margins, defects in photosynthetic activity and altered plant morphology (Marschner, 1995; Kopsell and Kopsell, 2007). Furthermore, because Cu is not a mobile element, it is not efficiently redistributed from old leaves (OL) to young leaves (YL), meristems, and reproductive organs. Therefore deficiency symptoms manifest themselves first in young developing tissues resulting also in reduced fertility (Marschner, 1995; Kopsell and Kopsell, 2007). Symptoms of Cu deficiency can be species-specific and often depend on the stage of deficiency (Kopsell and Kopsell, 2007). Cereals can undergo lodging while in trees wood quality and production can be severely affected during Cu deprivation (Ruiter, 1969; Voelker et al., 2010). In the poplar, *Populus trichocarpa*, Cu depletion strongly decreased the activity of several Cu proteins including PC, which resulted in decreased photosynthesis and ultimately decreased plant growth (Ravet et al., 2011). However, in *P. trichocarpa* the PC mRNA levels were only slightly decreased in Cu deprived plants and sufficient PC protein remained active so that a basal level of photosynthesis could be maintained (Ravet et al., 2011). Indeed upon Cu resupply these plants recovered very quickly to full photosynthetic capacity. In contrast, Cu depletion very strongly affected the expression of other Cu proteins (CSDs, PPO) via Cu miRNAs (Ravet et al., 2011). These findings in *P. trichocarpa* suggested that Cu-miRNAs indeed help to prioritize the use of Cu and thus support the above-mentioned Cu economy model (Ravet et al., 2011).

*Populus trichocarpa* shows an atypical growth in hydroponics; its stems grow vertically at a high pace with leaves expanding rapidly to full size resulting in a high but relatively open canopy. Another variety of poplar, the hybrid white poplar (*Populus tremula* x *P. Alba*, INRA 717-1B4) shows a more typical growth pattern in hydroponics, with leaves that expand more gradually and for a longer time, thus forming a much denser canopy especially toward the lower part of the stem. Overall, this hybrid poplar forms photosynthetic leaf surface even more rapidly than *P. trichocarpa*. In addition, hybrid poplar can be transformed efficiently, something which is harder to do with *P. trichocarpa* making further analysis of the Cu-economy model more challenging in the latter variety. Fortunately, the full genome sequence that is available for *P. trichocarpa* (Tuskan et al., 2006) can be used as a reliable scaffold for genomic tool development in hybrid poplar. Responses to dynamic changes in Cu availability have not been described for hybrid poplar. Because we are especially interested in Cu homeostasis in the context of photosynthesis we aimed to analyze the response of the photosynthetic machinery to varying Cu supply in hybrid poplar. We compared physiological, biochemical, and molecular responses including previously not described mRNA and microRNA expression patterns between older and YL which revealed unexpected and novel regulation of Cu homeostasis superimposed upon the Cu-microRNA mediated Cu economy model.

## Materials and Methods

### Plant Material and Growth Conditions

Hybrid white poplar plants (*P. tremula* x *P. Alba*, INRA 717-1B4) were propagated in a plant growth room from greenwood cuttings collected from 3- to 6-months-old trees grown in soil (Fafard 4P Mix Professional Formula 2.8 CU. FT. (79.3 L); Sun Gro Horticulture, Agawam, MA, USA). For hydroponic culture, we used 8-cm-tall apical stem cuttings (one per branch). Cuttings made from the same plant were randomly distributed over treatments. An indole butyric acid-containing mixture (Clonex; Hydrodynamics International, Lansing, MI, USA) was used to initiate roots from cuttings which were kept in vermiculite under saturated humidity. After rooting (approximately 10 days), the clones were transplanted into 20-L black plastic buckets (three plants per bucket) containing aerated one-tenth-strength modified Hoagland’s solution (Hoagland and Arnon, 1950). The final solution consisted of 0.13 mM NH_4_NO_3_, 0.53 mM Ca(NO_3_)_2_.4H_2_O, 0.68 mM KNO_3_, 0.19 mM MgSO_4_.7H_2_O,0.1 mM KH_2_PO_4_, 4.5 μM Fe^3+^-EDTA, 0.33 μM MgCl_2_.6H_2_O, 0.61 μM H_3_BO_3_, 0.12 μM MnSO_4_.H_2_O, 0.01 μM ZnSO_4_.7H_2_O, 0.002 μM MoO_3_ with or without 50 nM CuSO_4_.5H_2_O. The media were adjusted to pH 5.9. The nutrient solution was aerated continuously and replaced weekly. For growth under Cu depletion, Cu was omitted from the nutrient solution, while CuSO_4_ was added for final 50 nM concentration for the Cu-sufficient condition and for the Cu-resupply experiments. To minimize Cu contamination, deionized water was used and buckets used for Cu-deprivation had never seen Cu. Plants were grown in a climate controlled room under light intensity of 150 μmol m^-2^ s^-1^, with a 16-h-light/8-h-dark cycle and temperature maintained at 22°C ± 1°C.

### Plant Sampling and Plant Height Measurements

All experiments were performed with at least three biological replicates. Sampling for elemental analysis, RNA isolation, and protein extraction was partitioned into young leaves (YL; LPI 0–2) and mature, older leaves (OL; LPI 3–9; Larson and Isebrands, 1971). Material from three to four plants was pooled into a single sample. Samples were either immediately frozen in liquid nitrogen and stored frozen at -80°C until further analysis for protein or RNA analyses, or they were dried for elemental analysis. For chlorophyll fluorescence measurements, YL (LPI 2) and mature leaves (LPI 5–6) were studied. Plant height was measured from the top of the buckets to the end of the stem every week.

### Elemental Analysis

For Cu and mineral nutrient content analysis, roots were rinsed in deionized water (for 3 s × 30 s) at the time of harvest. Plant material was dried for 72 h at 55°C. Hundred-milligram of dried tissue was digested in 1 mL of concentrated nitric acid and heated at 60°C for 2 h and at 130°C for 6 h. Resulting digests were diluted up to 10 mL with double-distilled water and analyzed using inductively coupled plasma atomic emission spectrometry (ICP-AES) as described by Pilon-Smits et al. (1999).

### Chlorophyll Fluorescence Measurements

All plants were dark adapted for 1 h prior to analysis. Intact young and mature leaves were used. For the analysis of Cu depleted and control plants after 5 weeks of treatment, chlorophyll fluorescence was measured using an FMS system (Hansatech, Norfolk, UK) as previously described (Cohu and Pilon, 2007; Ravet et al., 2011). For comparison of plants before and after Cu-resupply we used a Dual PAM 101 with software version 1.18 (Heinz Walz, Effeltrich, Germany). Chlorophyll fluorescence was analyzed at 22°C, the indicated actinic light (red LED) settings were used with a 30 s wait between saturating pulses. All parameters were calculated according to Klughammer and Schreiber (2008).

### Protein Accumulation

Soluble proteins for SDS-polyacrylamide gel analysis were extracted as described (Abdel-Ghany et al., 2005). Protein concentration was determined according to Bradford (1976) using bovine serum albumin as standard. Each experiment was done at least in biological triplicate with identical results, and the representative gels are shown. For western blotting, 30 μg of total protein was separated by 15% SDS-PAGE and then transferred onto a nitrocellulose membrane. Antibodies used for immunodetection of PC, CSD1, CSD2, and cFBPase have been described (Cohu et al., 2009; Ravet et al., 2011). For SOD activity, total soluble proteins (30 μg) was fractioned on a non-denaturing 15% acrylamide gel and stained for total SOD activity as previously described (Beauchamp and Fridovich, 1971); specific SOD isoforms were identified based on their sensitivity to inhibitors and their mobility on native gels (Abdel-Ghany et al., 2005; Ravet et al., 2011). For the in-gel PPO activity assay, 30 μg of native protein was fractioned by SDS-PAGE (10% gel) and stained for PPO activity using the L-3,4-dihydroxy-Phe method as described in Constabel et al. (2000).

### Quantitative Reverse Transcription-PCR

Total RNA was extracted using Trizol reagent (Life Technologies, Carlsbad, CA, USA) according to the manufacturer’s recommendations. Total RNA concentration was determined and equal amounts per sample were reverse transcribed with random hexamer primers using the First Strand cDNA Synthesis Kit (Life Technologies). Quantitative RT-PCR was performed using the Light Cycler SYBR Green l master mix (Life Technologies). Samples without template were used as negative controls. PCa/b, CSD1a/b, CSD2a/b, PPO10/12, and Actin 1 gene transcript abundance were analyzed using gene-specific primer pairs as described previously for *P. trichocarpa* (Ravet et al., 2011). New primers were designed specifically for FeSOD and LAC12/40 gene transcripts. All primers are listed in **Table 1**. Gene expression was monitored in biological triplicate, and the results were standardized using Actin1 as a housekeeping gene (Ravet et al., 2011). qRT-PCR results and quality controls were analyzed using Light-Cycler 480 data-analysis software (version 1.5.1, Roche, Basel, Switzerland). Relative transcript levels were calculated using the ΔΔCt method.

**Table 1:** List of the primers used qRT-PCR and mature miRNA stem-loop qRT-PCR.

qRT-PCR primers		
	**Sequence (5**′**to 3**′**)**	**Sense**
PCa/b	CATTGGCTTTTGTTCCCAGCG	Forward
	GGCAACTTCGAAAGTCTCTCC	Reverse
PCa	GTAACAAAGGCGAATACAGC	Forward
	CATATATTCTCTCATTCCCACC	Reverse
CSD1a	ATGATGGCACTGCTACTTTCA	Forward
	TCCCTTGCCAAGATCATCAG	Reverse
CSD1b	TGATGGCACTGCTACTGTCT	Forward
	TCCCTTGCCAAGATCATCAG	Reverse
CSD2a	CACTGAGTGGTCCAAATGCA	Forward
	GTTGAGCTTAGTTCATGCCC	Reverse
CSD2b	CACTGAGTGGTCCTAACACG	Forward
	GTTGAGCTGAGTTCATGCTT	Reverse
FeSOD	CCTCCATATCCCATGAATGC	Forward
	CTGTGCAGCGTTGTTGAAAG	Reverse
LAC12	CAAACCGTTCACCACATCAG	Forward
	ATGAACAATTCTTGGCAGCAC	Reverse
LAC40	GGCGGTTTCACTTTGCCAG	Forward
	GTGGCATCAACTTCCACGAC	Reverse
PPO10	GGACCTGAAGACCAAGTTCAC	Forward
	TGCCAGGCCCATTGCAAGAC	Reverse
PPO12	CACCCTGATTGGCTCGACG	Forward
	TATCTACTGGCACTGTCGGC	Reverse

**miRNA Stem-Loop qRT-PCR primers**

	**Sequence (5**′**to 3**′**)**	**Sense**

**Reverse transcription**		
miR397-RT	GTCGTATCCAGTGCAGGGTCCGAGGTATTCGCACTGGATACGACCATCAA	Reverse
miR398bc-RT	GTCGTATCCAGTGCAGGGTCCGAGGTATTCGCACTGGATACGACCAGGGG	Reverse
miR408-RT	GTCGTATCCAGTGCAGGGTCCGAGGTATTCGCACTGGATACGACGCCAGG	Reverse
miR1444a-RT	GTCGTATCCAGTGCAGGGTCCGAGGTATTCGCACTGGATACGACGAACAT	Reverse
miR156a-g	GTCGTATCCAGTGCAGGGTCCGAGGTATTCGCACTGGATACGACGTGCTC	Reverse
**qRT-PCR**		
miR397a	GTGTGTCATTGAGTGCAGCG	Reverse
miR397b	GTGTGCCATTGAGTGCAGCG	Reverse
miR398b/c	TGGGTGTGTTCTCAGGTCG	Reverse
miR408	GTGTGATGCACTGCCTCTTC	Reverse
miR1444a	TCCGCTCCACATTCGGTCA	Reverse
miR156a-g	ATGCGCTGACAGAAGAGAGT	Reverse
miRuniversal	CCAGTGCAGGGTCCGAGG	Forward

### Mature miRNA Stem-Loop qRT-PCR

For the stem-loop pulsed RT, total RNA was extracted as described above, however, ethanol washes were avoided and nucleic acid precipitation steps were performed using 1:1 (v/v) isopropanol and 1:10 (v/v) sodium acetate (3 M; pH 5.2) in order to optimize small RNA molecule retrieval. The stem-loop pulsed RT and miRNA qRT-PCR were performed as described previously (Varkonyi-Gasic and Hellens, 2007). miRNA397a/b, 398b/c, 408, and 1444a abundance were analyzed using gene-specific primers as described previously for *P. trichocarpa* (Ravet et al., 2011; see **Table 1**). qRT-PCR procedures and analysis of the data were performed as described above. Mature miRNA abundance was monitored in biological triplicate for each sample, and the results were standardized using miR156 expression (Ravet et al., 2011).

### Statistical Analysis

JMP software (version 9.0.2; SAS Institute, Cary, NC, USA) was used for statistical analysis. Results shown represent averages and SD from at least three independent biological replicates. A Student’s *t*-test was used to calculate significant differences (*p* < 0.05), which is reported in the text or figures where appropriate.

## Results

**FIGURE 1:**
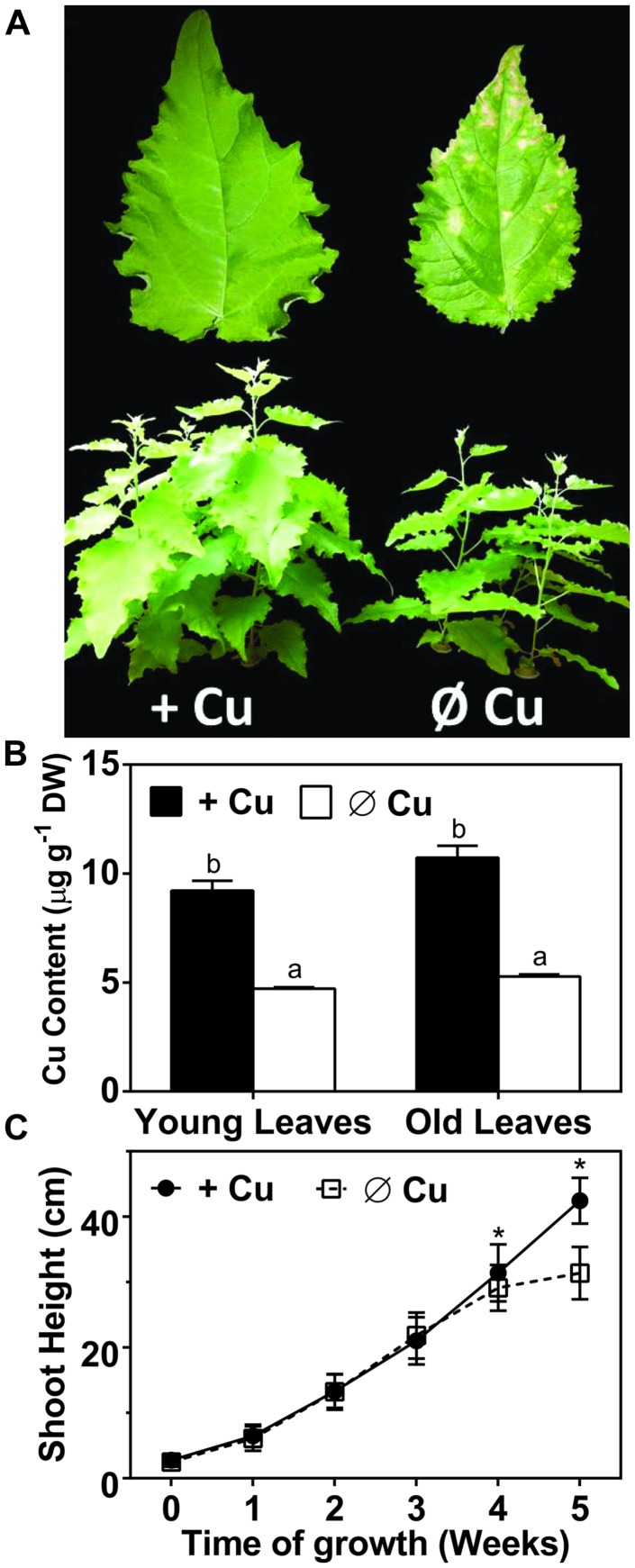
**Effects of copper (Cu) deprivation on plant growth, Cu content and shoot height of hybrid poplar.** Hybrid poplar cuttings were grown on 10% Hoagland solution containing 50 nM CuSO_4_ (+Cu) or 0 nM CuSO_4_ (Ø Cu). **(A)** First fully developed leaves and 5-weeks grown plants under Cu sufficient and Cu deprived conditions. **(B)** Copper content (μg g^-1^ DW) of young and old leaves (OL) was measured by inductively coupled plasma atomic emission spectrometry (ICP-AES). Values are given as averages ±SD (*n* = 3). **(C)** Growth was monitored by measurement of shoot height. Values are given as averages ±SD (*n* = 12). Different letters indicate significant differences within organs between treatments (^∗^*p* < 0.05, Student’s *t*-test).

A hydroponic system was used in order to characterize the symptoms caused by Cu deficiency over a period of several weeks. The 50 nM CuSO_4_ concentration in the control medium was previously shown to be sufficient for a range of plants (Cohu and Pilon, 2007) and allowed for vigorous growth of hybrid poplar (**Figure 1A**). Plants that were grown on Cu deprived medium for over 3 weeks started to show visible deficiency symptoms, including reduced biomass and browning in leaves (**Figure 1**). After 5 weeks the Cu content in the leaves was reduced to about 50% of the control for both young developing leaves and mature leaves (**Figure 1B**). Hybrid polar plants grown in Cu sufficient control conditions kept growing vigorously for over 5-weeks (**Figures 1A,C**). In contrast, plant height was affected by Cu deficiency already after 3-weeks, when growth started to slow down. Growth eventually ceased after 4-weeks for Cu deficient plants (**Figure 1C**). The symptoms of Cu deficiency were largely reversible if Cu was re-supplied at week 5, with brown spots turning green again except at the tip of the leaf. However, plants grown under continued Cu deficiency for over 6 weeks started to show severe and irreversible symptoms including curling, desiccation, and excessive browning of leaves (not shown). In conclusion, Cu deprivation led to a strong growth inhibition of hybrid poplar but the symptoms were largely reversible if Cu was given back before 6 weeks of depletion.

**FIGURE 2:**
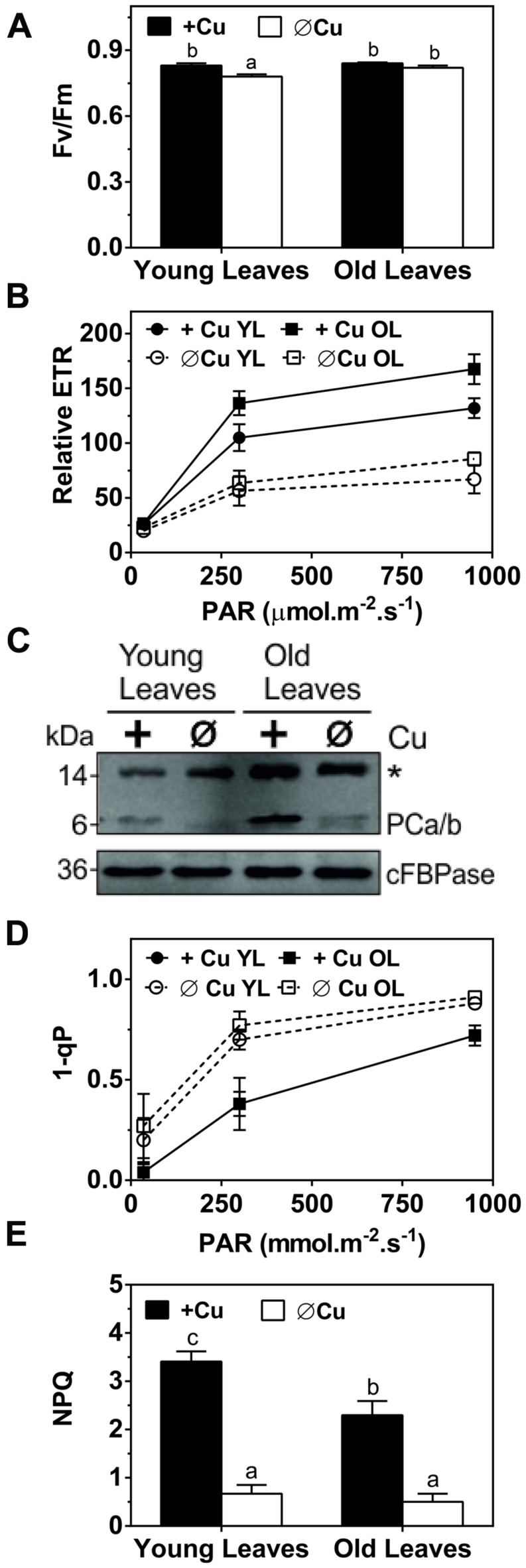
**Effects of Cu deprivation on photosynthetic parameters and on the plastocyanin (PC) abundance. (A)** Immunoblot of PC (PCa/b) for young leaves (YL) and OL. Total soluble proteins (30 μg) were fractioned by SDS-PAGE (15% gel) and blotted onto membranes. cFBPase was used a loading control. The asterisk (^∗^) indicates non-specific cross-reactivity of the PC antibody. **(B)** The maximum quantum yield of photosystem-II (Fv/Fm) of dark adopted YL and OL. **(C)** Response curve of the relative ETR to light intensities ranging from 35 to 950 μmol m^-2^s^-1^ in YL and OL. **(D)** Redox state of plastoquinone (1-qP) at light intensities ranging from 35 to 950 μmol m^-2^s^-1^ in YL and OL. **(E)** Non-photochemical quenching (NPQ) of dark-adapted YL and OL measured at 950 μmol m^-2^s^-1^. All values are given as averages ±SD (*n* = 5). Different letters above bars (in **A,D**) represent significant differences (*p* < 0.05, Student’s *t*-test).

Plastocyanin is the only Cu-containing protein directly involved in photosynthesis and it functions in the light reactions. To assess the capacity of the light reactions we compared chlorophyll fluorescence parameters for control and Cu depleted plants after 5 weeks of treatment (**Figure 2**). The parameter F_V_/F_M_, the maximum quantum yield of photosystem-II, is an indicator of photo-inhibition and therefore of stress to the photosynthetic machinery. There was a significant but small Cu-induced change in F_V_/F_M_ in younger leaves but not in older leaves (**Figure 2A**). In contrast, the ETR and 1-qP, which indicates the redox state of the plastoquinone pool, were both strongly affected by Cu depletion especially in younger leaves at all light intensities that were tested (**Figures 2B,D**). The PC content was analyzed by western blotting (**Figure 2C**). Interestingly, compared to mature leaves the younger leaves have a lower PC content even under control conditions. Under Cu-deprivation PC protein dropped to the detection limit in the YL (**Figure 2C**). NPQ, the dissipation of excess excitation energy in PSII as heat, which depends on acidification of the thylakoid lumen and thus upon electron transport, is indicated by the parameter NPQ. NPQ was also strongly diminished after Cu depletion (**Figure 2E**), which further indicates a defect in electron transport. We conclude that Cu deprivation caused a strong reduction in electron transport especially in younger leaves. The reduction in electron transport capacity upon Cu depletion can be ascribed to a strong loss of PC protein. In mature leaves, which have a higher PC content in the control condition, depletion of Cu does not lead to full depletion of PC and therefore some ETR capacity is maintained.

**FIGURE 3:**
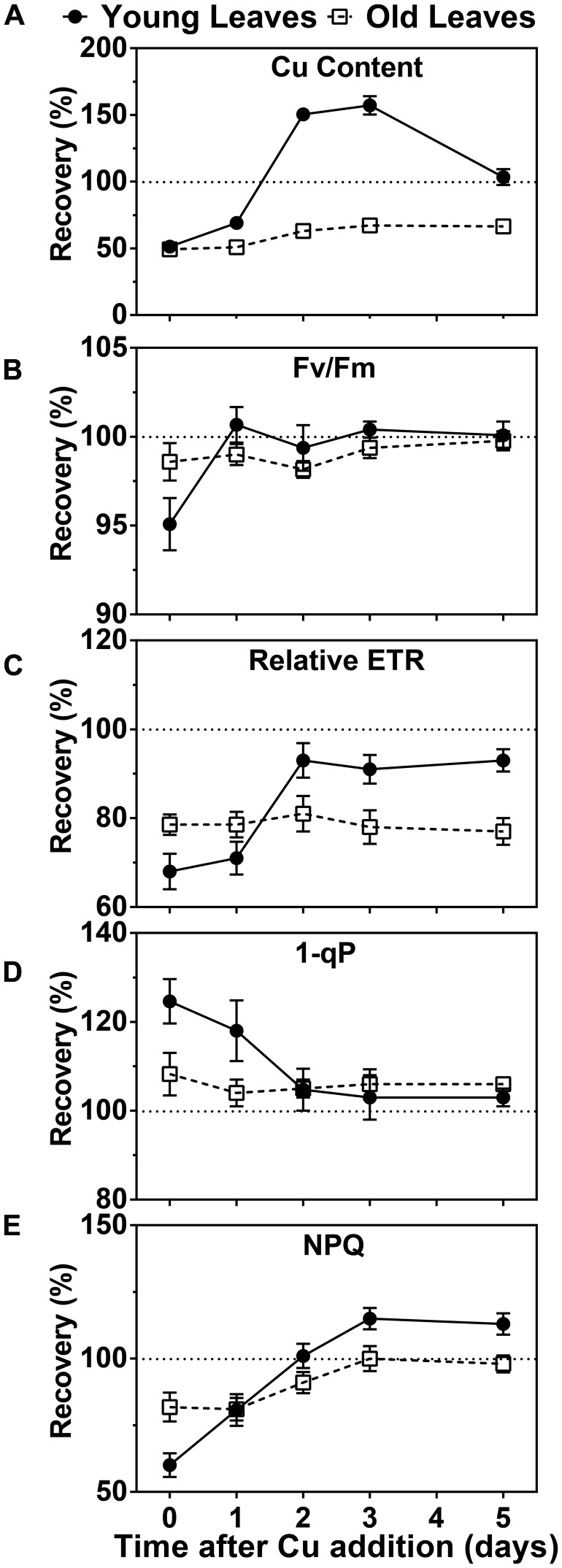
**Effects of Cu resupply to Cu-starved plants on Cu content and photosynthetic parameters.** Hybrid poplar cuttings were grown hydroponically in the absence of Cu as described in **Figure 1**. After 5 weeks, 50 nM Cu was added to the nutrient solution of plants previously grown in the absence of Cu. Plants continuously grown with 50 nM CuSO_4_ (+) or without added Cu (-) were used as control. Cu content and photosynthetic parameters before (0) and 1, 2, 3, and 5 days after Cu addition were calculated relative to the value obtained for the Cu-sufficient plants and are presented as percentages. **(A)** Cu content recovery in young and OL. Cu content was measured by ICP-AES. Values are given as averages ±SD (*n* = 6). **(B–E)** Recovery of Fv/Fm, relative ETR, 1-qP and NPQ. Photosynthetic parameters were measured at 1095 μmol m^-2^s^-1^. Values are given as averages ±SD.

Since symptoms of Cu deficiency were largely reversible by Cu-resupply at week 5 we were prompted to investigate the kinetics of this recovery at the physiological and molecular level. We first measured the Cu content in photosynthetic tissue after Cu was added back to the growth medium of hybrid poplar that had been depleted for 5 weeks. A rise in the Cu concentration was already observed after 1 day in the YL. Two and three days after Cu resupply, the Cu levels in YL were above the levels observed in control plants while at day 5 the Cu content returned to control levels of about 10 ppm (**Figure 3A**). In contrast, Cu levels in the older leaves of Cu-starved plants came up only slightly after resupply and control levels of 12 ppm were not reached (**Figure 3A**). We also characterized the recovery of chlorophyll fluorescence parameters (**Figures 3B–E**). The parameter F_V_/F_M_ recovered within a day for YL and was stable in the older leaves. The parameters ETR, as well as 1-qP both started to recover after a day and were fully back to control values after just 2 days in the YL, whereas these parameters did not recover for the older developed leaves within the test period. Similarly, the NPQ capacity was also recovered after 2 days in the YL but its recovery lagged more in the older leaves. In conclusion, upon Cu resupply to previously depleted plants, Cu delivery is prioritized to the younger developing leaves with a concomitant recovery of photosynthetic capacity.

**FIGURE 4:**
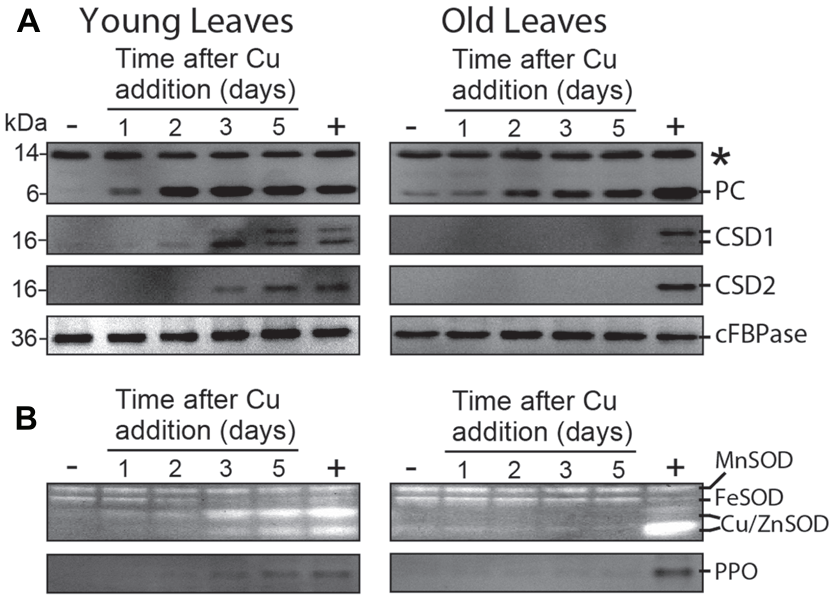
**Effects of Cu resupply to Cu-starved plants on the abundance and activity of Cu proteins. Hybrid poplar cuttings were grown hydroponically in the absence of Cu as described in **Figure 1**.** After 5 weeks, 50 nM Cu was added to the nutrient solution of plants previously grown in the absence of Cu. Plants continuously grown with 50 nM CuSO_4_ (+) or without added Cu (-) were used as control. **(A)** Immunoblots for PC, the cytosolic (CSD1) and the plastidial (CSD2) Cu/ZnSODs. Cytosolic Fru-1,6-biphosphate (cFBPase), which is not affected by Cu status (Ravet et al., 2011; Tapken et al., 2012), was used as loading control. Total soluble proteins (30 μg) were fractioned by SDS-PAGE (15% gel) and blotted onto membranes. The asterisk (^∗^) indicates non-specific cross-reactivity of the PC antibody. cFBPase was used a loading control. **(B)** For SOD activity, total soluble proteins (30 μg) was fractioned on a non-denaturing 15% acryl amide gel and stained for total SOD activity. For the in-gel polyphenol oxidase (PPO) activity assay, 30 μg of native protein was fractioned by SDS-PAGE (10% gel) and stained for PPO activity using the L-3,4-dihydroxy-Phe method. All images of immunoblots and activity gels are representative of at least three repeats.

The different recovery of electron transport in the younger developing leaves versus older leaves, prompted us to analyze changes in PC levels in these organs by immunoblotting. Like most plants, hybrid poplar has two isoforms of PC; in hybrid poplar and in *P. trichocarpa* (Ravet et al., 2011) the two PC isoforms have the same mobility on SDS-PAGE (**Figure 4**). The PC content of YL was below detection level at the start of resupply. Within a day of Cu resupply the PC levels in these leaves were starting to recover with full recovery after 2 days (**Figure 4A**; Supplementary Table S1). In comparison to the younger leaves, PC content recovered much slower in the older leaves. Overall, the recovery of PC parallels the recovery of electron transport related chlorophyll fluorescence parameters and Cu content (**Figure 3**). For comparison we also analyzed superoxide dismutase isoforms. Native gel and immunoblot analyses indicated that isoforms of cytosolic CuZnSOD (CSD1) were differentially expressed in YL versus OL (**Figure 4**). In the YL a CSD1 isoform with higher mobility on SDS-PAGE was the dominant form, whereas in older leaves an isoform with lower mobility dominated (**Figure 4A**). Nevertheless and as expected, both the cytosolic (CSD1) and chloroplastic (CSD2) isoforms were strongly affected by Cu depletion. In the younger leaves where Cu levels came up very rapidly (**Figure 3A**) CSD isoform recovery was clearly much slower and lagged 2 days when compared to PC (**Figure 4A**). Strikingly, and surprisingly, in older leaves both CSD1 and CSD2 remained below detection level after Cu starvation even 5 days after Cu resupply. Native gel analysis of SOD isoform activities for both young and older recovering leaf samples confirmed the immunoblot results for Cu/ZnSOD. In addition these native gels showed that FeSOD activity was very low in control (Cu-sufficient) plants. However, FeSOD activity was high in Cu-starved leaves. This FeSOD activity diminished around day 3 of Cu resupply in younger leaves but remained unaffected by Cu resupply in the older leaves.

Cu/ZnSODs are targets of miR398, one of the Cu-microRNAs. To investigate if the delayed recovery of the Cu/ZnSODs was representative of other Cu-microRNA targets we also analyzed PPO, which accumulates in the thylakoid lumen and is a target of miR1444. PPO activity was very low after Cu starvation but recovered with kinetics similar to Cu/ZnSOD in younger leaves and never came back with the 5 days recovery period in the older leaves.

**FIGURE 5:**
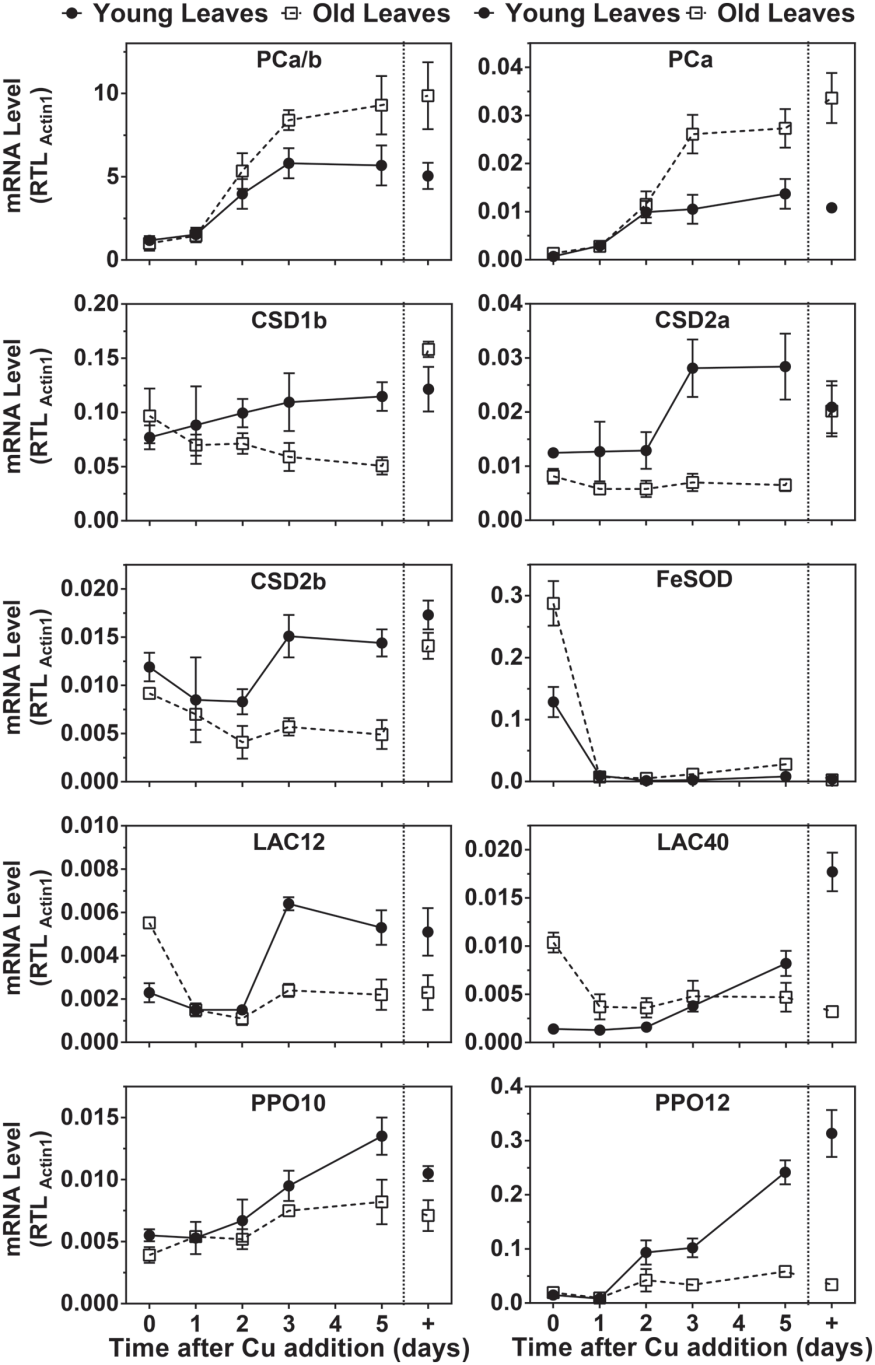
**Effects of Cu resupply to Cu-starved plants on RNA expression level of Cu proteins.** Hybrid poplar cuttings were grown hydroponically in the absence of Cu as described in **Figure 1**. After 5 weeks, 50 nM Cu was added to the nutrient solution of plants previously grown in the absence of Cu. Plants continuously grown with 50 nM CuSO_4_ (+) were used as control. Total PC (PCa/b), PCa, CSD1b, CSD2a, CSD2b, FSD2, LAC12, LAC40, PPO10, and PPO12 relative transcripts levels (RTL) were determined using qRT-PCR. Values are normalized relative to Actin1 expression and are given as averages ±SD (*n* = 3).

Because PC protein levels were strongly affected by the Cu treatments we also analyzed PC mRNA abundance and compared it to transcripts that were known to be Cu-regulated (**Figure 5**). The two PC genes in hybrid poplar are not expressed at the same level, unlike the situation in *P. trichocarpa* (Ravet et al., 2011). However, the patterns of the responses to Cu seemed similar for both PC transcripts. Under control conditions, the PC transcript levels were roughly 1.5 times higher in older leaves compared to developing leaves, which reflects also the relative PC protein levels in these organs. Surprisingly, the abundance of both poplar PC transcripts was strongly decreased by Cu depletion in both YL and OL. It should be noted, however, that the PC transcript levels were maintained at roughly 10–20% of control levels after depletion. Upon resupply, the PC transcripts quickly came up and reached control levels in about 3 days.

In the related *P. trichocarpa* we previously detected two Cu/ZnSOD isoforms closely related to the *Arabidopsis* cytosolic CSD1, and for two isoforms closely related to chloroplastic CSD2 (Ravet et al., 2011). In hybrid poplar, the protein accumulation of CSD1 and CSD2 isoforms was strongly affected by Cu depletion (**Figure 5**). Transcript levels for CSD1 and CSD2 isoforms also significantly decreased in leaves upon Cu depletion. Furthermore, CSD2 mRNA decreased in both YL and OL in Cu deficient plants. However, it is important to note that all these Cu/ZnSOD transcripts remained detectable in all conditions. Upon Cu resupply an increase was seen in the Cu/ZnSOD transcripts in YL albeit that compared to PC mRNA the CSD1 and CSD2 mRNA levels took longer to recover. No recovery of Cu/ZnSOD transcripts was observed for OL and CSD2b mRNA even kept declining after Cu resupply. The major FeSOD in the plastids of *Arabidopsis* and other plants is well know to be induced by Cu depletion (Cohu and Pilon, 2007; Abdel-Ghany and Pilon, 2008). However, in *P. trichocarpa* no FeSOD activity had been detected (Ravet et al., 2011). Because we did observe a Cu-regulated FeSOD activity in hybrid poplar (**Figure 4B**) we investigated FeSOD transcript levels which revealed strong regulation by Cu.

In the poplar genome there are several LAC and PPO sequences annotated which show tissue specific expression as well as Cu-regulation (Tuskan et al., 2006; Ravet et al., 2011). We analyzed transcript levels of two LAC and two PPO isoforms that have abundant expression in leaves. The transcript levels of PPO10 and especially PPO12 decreased in young and OL in Cu deficient plants (**Figure 5**). Both transcripts show a recovery after Cu resupply with a lag of 2 days. The strongest recovery is seen in the younger leaves. LAC gene expression in response to Cu was different for young and OL. In younger leaves, Lac12 and 40 expression was reduced in Cu starved plants but it recovered upon resupply with a 2 days lag period, similar to CSD mRNA. In stark contrast, in the older leaves LAC12 and LAC40 mRNA levels were elevated on low Cu but came down with Cu-resupply. Overall mRNA expression patterns of the genes we investigated show unanticipated leaf age specific responses to Cu-depletion. Unexpectedly, PC mRNA is strongly Cu regulated but the dynamics of the PC mRNA changes are distinct from the genes that encode other Cu proteins.

**FIGURE 6:**
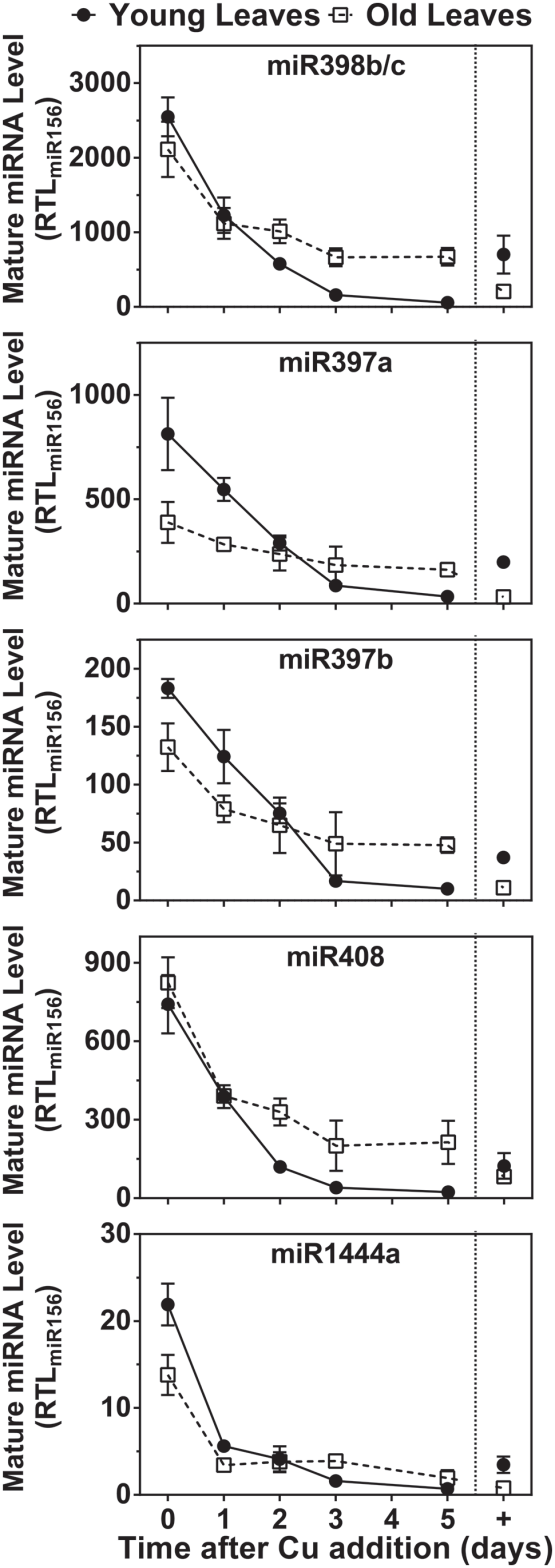
**Effects of Cu resupply to Cu-starved plants on Cu-miRNA expression level.** Hybrid poplar cuttings were grown hydroponically in the absence of Cu as described in **Figure 1**. After 5 weeks, 50 nM Cu was added to the nutrient solution of plants previously grown in the absence of Cu. Plants continuously grown with 50 nM CuSO_4_ (+) were used as control. Mature miR398b/c, miR397a, miR397b, miR408, and miR1444a RTL were determined using qRT-PCR. Values are normalized relative to miR156 expression and are given as averages ±SD (*n* = 3).

Because mRNAs for Cu/ZnSODs, several LACs and PPO are targets for Cu microRNAs, we also investigated expression patterns of miR398 (which targets Cu/ZnSOD), miR397, and miR408 (which target LACs) and miR1444 (which targets PPO). The results are shown in **Figure 6**. All Cu microRNAs show a comparable expression pattern resembling FeSOD mRNA. In the control condition, Cu-microRNA expression is low but slightly elevated in younger leaves, which also showed lower PC accumulation and thus seem slightly Cu deprived in this condition. Expression of Cu-microRNAs is induced by Cu depletion and expression drops within a day of Cu resupply. Older leaves maintain slightly higher Cu-microRNA expression after 5 days of recovery. A comparison of the data in **Figures 5** and **6** reveals that the expression of Cu protein mRNAs is not only a result of abundance of the matching, negatively regulating Cu-microRNAs.

## Discussion

Symptoms of Cu deficiency are well known to manifest in YL (Marschner, 1995; Burkhead et al., 2009). Since much of the Cu found in a plant’s areal tissues is in the chloroplasts in PC, we were especially interested in the effects of Cu depletion on the light reactions of photosynthesis. The experimental setup allowed us to deplete plants of Cu so that strong symptoms were observed. Importantly, however, the lack of a major effect of Cu depletion on F_V_/F_M_ indicates that non-specific or secondary effects on the photosynthetic machinery are relatively minor after 5 weeks of depletion. Indeed, upon resupply the visible symptoms were largely reversible within 5 days and plants could continue to grow vigorously. In order to gain insight into the processes that underlie recovery after Cu resupply we compared the light reactions together with a molecular analysis of Cu-protein expression. A previous study in *P. trichocarpa* also compared the effects of depletion in different organs (Ravet et al., 2011). However, the latter study on *P. trichocarpa* did not include a comparison of transcripts and microRNAs for the resupply time course. Furthermore, when compared to *P. trichocarpa* the effects of Cu depletion in hybrid poplar on photosynthesis are stronger with more pronounced differences between OL and YL, which we ascribe to the difference in growth and development as hybrid poplar forms a broad canopy much faster.

**FIGURE 7:**
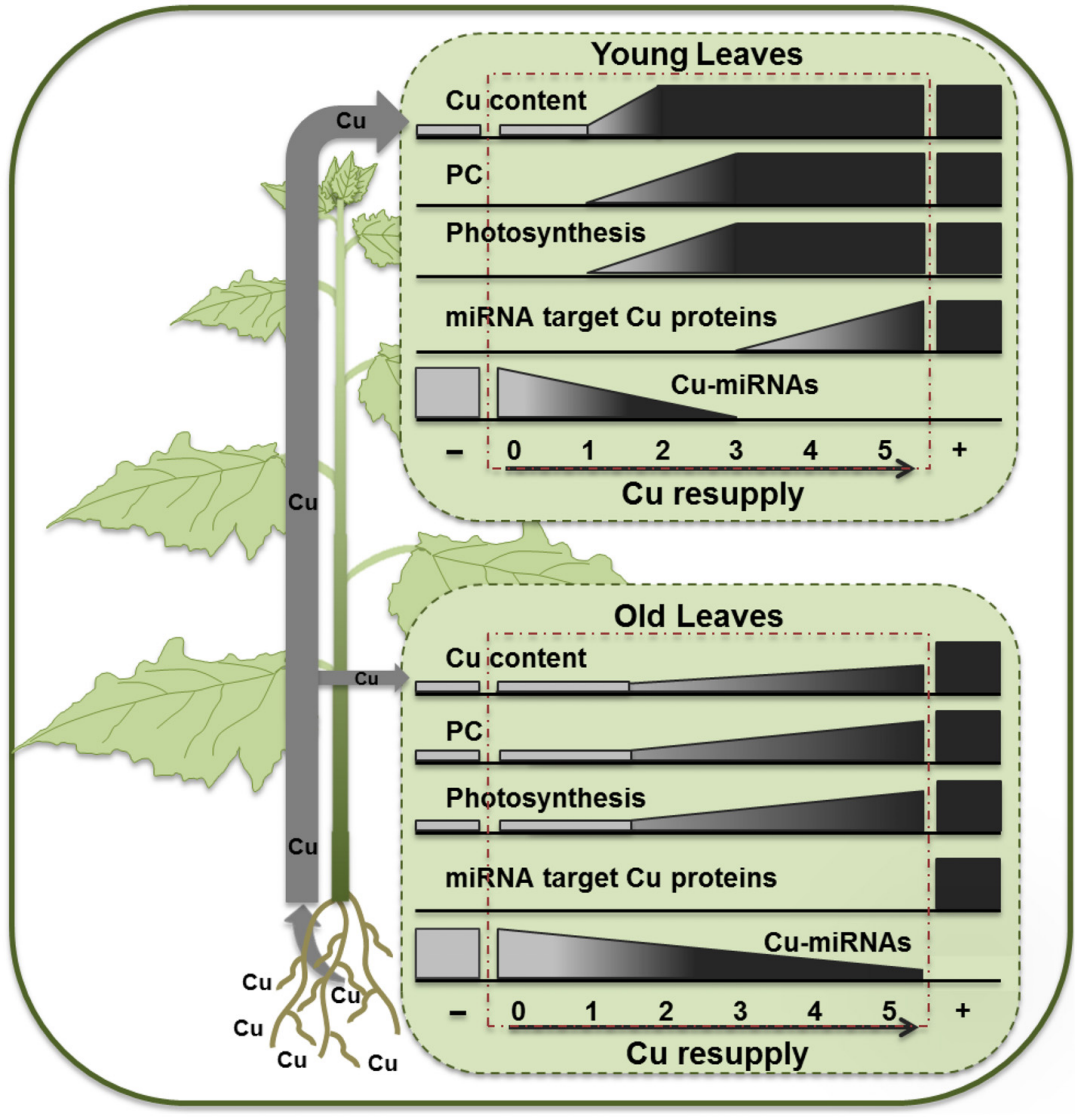
**Schematic diagram summarizing the effects of Cu depletion and resupply in young and OL of hybrid poplar**.

The observed results for hybrid poplar are schematically summarized in **Figure 7**. A 5-weeks long Cu depletion treatment caused Cu content to drop to about 50% of the control values for both younger and older leaves. However, the symptoms of Cu depletion differed between young, still developing, leaves and the mature leaves, not only at the physiological but also at the molecular level. Even more remarkable was the recovery after Cu resupply, for which younger and older leaves showed large differences as Cu came back quickly in YL but not in OL, revealing a clear priority favoring photosynthesis in YL. The recovery of PC protein upon Cu-resupply is also fast especially in YL, which can explain the fast recovery of ETR activity concomitant with Cu levels. The strong correlation of PC content and ETR agrees with studies of photosynthetic capacity acclimation in tobacco (Schöttler et al., 2004). In contrast, recovery of activity of Cu proteins that are Cu-microRNA targets is delayed. The Cu microRNAs become depleted only 3 days after Cu supply, and the expression patterns over the resupply time-course in the YL can fully explain the delay in Cu protein expression relative to PC. In *P. trichocarpa* two homologs, called *PtSPL3* and *PtSPL4,* are present of the *Arabidopsis* Cu-responsive master regulator *AtSPL7* (Lu et al., 2011). The observations for the YL can be largely explained within the context of a poplar *SPL3/4* mediated Cu economy model where the Cu-microRNAs mediate the down-regulation of redundant Cu-proteins (Lu et al., 2011; Ravet et al., 2011; Zhang et al., 2014).

The older leaves responded differently to Cu depletion and resupply. Firstly, the reduction in PC content upon Cu depletion was less when compared to younger leaves. The changes in CSD, LAC, and PPO proteins and their transcripts also differed between YL and OL with isoform-specific patterns of depletion and recovery. The OL and YL had comparable Cu contents of around 5 ppm upon Cu-depletion and therefore it may be hard to ascribe these differences to Cu contents only. It is important to note that the PC levels in control plants are much lower in the younger plants. It is reasonable to assume that these developing leaves still are building up their photosynthetic machinery and are getting ready to expand. It is therefore also reasonable to expect a much higher capacity to import Cu in the YL. The older leaves have had more time to accumulate Cu for use in PC and have a more established photosynthetic machinery which apparently can continue to function under Cu depletion even with some expansion of the leaf surface. It is perhaps likely that the older leaves, which seem too have acquired enough Cu to maintain a minimum photosynthetic activity, have a reduced capacity for Cu acquisition, which in turn would favor more Cu delivery to the younger leaves. Indeed, upon Cu resupply the YL show a fast rise in Cu content even over-shooting the control levels temporarily 2–3 days after Cu addition, before coming back to control levels (**Figure 3A**). At this point, it is not clear which molecular mechanism would be responsible for the lower Cu-acquisition in older leaves and the direction of Cu to younger leaves. Cu ions should reach the older leaves with the transpiration stream via the xylem. From there, Cu ions may be re-directed via the phloem toward young developing leaves. In analogy to what was reported for rice plants (Zheng et al., 2012), a yellow stripe like-transporter might be active to load Cu into the phloem in the older leaves. In addition, low activity of mesophyll cell surface COPT family transporters that mediate cellular Cu uptake (Sancenon et al., 2003) might limit Cu uptake in the older leaves. With their photosynthesis still active and growth in younger leaves limited by Cu, the OL maintain growth even after Cu depletion. This sustained growth during the depletion will further dilute the available Cu in older leaves. Together with a lack of Cu acquisition capacity this should help to maintain Cu-microRNA expression at a relatively high level (**Figure 6**), which in turn keeps Cu protein expression low in the older leaves (**Figures 4** and **5**). In turn this could make the older leaves a weaker sink for Cu, relative to the younger leaves.

We observed strong decreases in the PC protein levels after Cu depletion especially in younger leaves. These finding agree with observations made before in *Arabidopsis* (Abdel-Ghany and Pilon, 2008) and in *P. trichocarpa* (Ravet et al., 2011). However, in hybrid poplar the mRNA levels for PC also strongly responded to Cu with a very large reduction in PC transcripts for depleted plants followed again by a very strong recovery upon resupply (**Figure 5**). For the PC transcripts the older leaves showed the strongest response. These very large changes in PC mRNA levels in response to Cu were unexpected in view of previous observations in *Arabidopsis* (Abdel-Ghany and Pilon, 2008; Bernal et al., 2012) and in *P. trichocarpa* (Ravet et al., 2011). In *Arabidopsis*, PC1 and PC2 mRNA levels were not affected by Cu depletion treatments, not in seedlings and not in mature plants (Abdel-Ghany and Pilon, 2008; Bernal et al., 2012). In *P. trichocarpa*, a significant yet relatively small reduction in PC transcript levels (to 70% of control levels) was only seen for YL. There is no microRNA target site predicted in the *P. trichocarpa* PC sequences and we therefore can assume that PC is also not a microRNA target in the closely related hybrid poplar sequence. Therefore the strong effect of Cu on PC mRNA most likely involves transcriptional regulation that is more indirectly a consequence of Cu depletion. PC expression in *Arabidopsis* requires light and a green chloroplast suggesting that control of PC expression involves positive feedback (Vorst et al., 1993). The lack of Cu in hybrid poplar leaves might cause a partial disruption in the positive feedback loop; as PC protein which fails to mature cannot contribute to the photosynthesis- and plastid-derived signals required for the full expression of PC and perhaps other photosynthesis related proteins.

The relatively high capacity of hybrid poplar to rapidly expand its canopy, could effectively keep available Cu at a low threshold causing low PC expression. In *P. trichocarpa* and *Arabidopsis* these thresholds are perhaps not reached as quickly. As a consequence of its capacity to form photosynthetic leaf surface very rapidly, hybrid poplar new leaf development might be placed in a holding pattern upon Cu depletion as the tree sustains itself on photosynthesis in its older leaves. As Cu becomes available, however, it is delivered rapidly to the new developing leaves, which will soon form the next layer at the top of the canopy performing most of its photosynthesis.

## Conflict of Interest Statement

The authors declare that the research was conducted in the absence of any commercial or financial relationships that could be construed as a potential conflict of interest.

## Abbreviations

Cu/ZnSOD: Cu/Zn-superoxide dismutase
ETR: relative electron transport rate
FeSOD: iron-superoxide dismutase
F_V_/F_M_: maximum quantum yield of photosystem II
LAC: laccase
NPQ: non-photochemical quenching
PC: plastocyanin
PPO: polyphenol oxidase
OL: older leaves
YL: young leaves
