# Supplementary material for: Prioritization of copper for the use in photosynthetic electron transport in developing leaves of hybrid poplar
Source: Front Plant Sci. 2015 Jun 3;6:407. doi: 10.3389/fpls.2015.00407 (PMC4452806; doi:10.3389/fpls.2015.00407)
Supplement: Supplementary file 1 [file Table_1.PDF]

Copper (Cu) content µg g^{-1} dry weight					
Time after Cu addition (days)	0	1	2	3	5
Young leaves	4.72 ± 0.06	6.37 ± 0.27	13.86 ± 0.67	14.46 ± 0.36	9.52 ± 0.45
Old leaves	5.28 ± 0.09	5.45 ± 0.14	6.75 ± 0.19	6.15 ± 0.34	6.17 ± 0.18

Supplementary table S1. Effects of Cu resupply to Cu-starved plants on Cu content of young and old leaves. Hybrid poplar cuttings were grown hydroponically in the absence of Cu as described in Figure 1. After 5 weeks, 50 nM Cu was added to the nutrient solution of plants previously grown in the absence of Cu. Cu content before (0) and 1, 2, 3 and 5 days after Cu addition are given in the above table. Cu content was measured by ICP-AES. Values are given as averages ± SD (n = 3).
